# Gastric Peroral Endoscopic Myotomy (G-POEM) as a Treatment for Refractory Gastroparesis: Long-Term Outcomes

**DOI:** 10.1155/2018/6409698

**Published:** 2018-10-22

**Authors:** Jiaxin Xu, Tianyin Chen, Shaimaa Elkholy, Meidong Xu, Yunshi Zhong, Yiqun Zhang, Weifeng Chen, Wenzheng Qin, Mingyan Cai, Pinghong Zhou

**Affiliations:** ^1^Endoscopy Centre and Endoscopy Research Institute, Zhongshan Hospital, Fudan University, Shanghai, China; ^2^Internal Medicine Department, Gastroenterology Division, Faculty of Medicine, Cairo University, Egypt

## Abstract

**Background and Aims:**

Gastric peroral endoscopic myotomy (G-POEM) has been regarded as a novel and minimally invasive therapy for refractory gastroparesis. This study reports the long-term outcomes and possible predictive factors for successful outcomes after G-POEM in an Asian population.

**Methods:**

This is a retrospective single-centre study of 16 patients who underwent G-POEM for refractory gastroparesis from August 2016 to October 2017. This study included 11 males and 5 females; in addition, 13 patients had postsurgical gastroparesis, and 3 patients had diabetes. The patients included had severe and refractory gastroparesis, as indicated by a Gastroparesis Cardinal Symptom Index (GCSI) score ≥20, and evidence of a delay on gastric emptying scintigraphy (GES). The primary outcome parameter was an assessment of the long-term clinical efficacy of the procedure. The secondary outcome parameter was the detection of possible predictive factors for success and the determination of cut-off values for such predictors.

**Results:**

Technical success was achieved in 100% of the patients, with a mean procedure time of 45.25±12.96 min. The long-term clinical response was assessed in all patients during a median follow-up of 14.5 months. Clinical success was achieved in 13 (81.25%) patients. There was a significant reduction in the GCSI scores and GES values after the procedure compared to the baseline values, with* P* values of <0.0001 and 0.012, respectively. Univariate regression analysis showed that the GCSI and GES had significant associations with the future clinical outcomes of the patients, but this finding was not confirmed in multivariate analysis. A GCSI cut-off score of ≤30 had a high sensitivity and a negative predictive value (NPV) of 100% for predicting a successful procedure. GES (half emptying time ≤221.6 min and 2-hour retention ≤78.6%) had a high specificity and a positive predictive value (PPV) of 100%.

**Conclusions:**

G-POEM is a safe and effective treatment option with a long-term efficacy of 81.6%. GCSI and GES could serve as good predictive measures.

## 1. Introduction

Gastroparesis is a chronic and debilitating gastric motility disorder with limited effective therapeutic options [1]. This disorder can result in frequent hospitalization and repeated nutrition support interventions. Multiple conditions have been associated with gastroparesis, and most aetiologies are postsurgical, diabetic, or idiopathic [2]. The pathogenesis of delayed gastric emptying is associated with fundus abnormalities, antrum and antroduodenal discoordination, pyloric dysfunction, and abnormal small bowel motility [3]. The pathogenesis of gastroparesis comprises two main components: altered gastric motility and increased pyloric pressure. Currently available medical therapies have limited efficacy and the potential for significant adverse events [4].

A recent innovation of the endoscopic submucosal tunnelling technique is gastric peroral endoscopic myotomy (G-POEM). This technique is emerging as a promising option for the treatment of refractory gastroparesis. In 2013, Khashab et al. [5] reported the first human case in a 27-year-old woman who was diagnosed with insulin-dependent diabetes mellitus at age 17 and was evaluated in the clinic for diabetic gastroparesis. Twelve weeks after the procedure, she remained well with significant improvement in daily symptoms and was able to tolerate a soft diet appropriately. Since then, several case reports [6, 7] and small case series [8, 9] have been published. Mekaroonkamol et al. [10] reported symptomatic improvement in patients with postinfection, postsurgery, and idiopathic gastroparesis. This technique is starting to attract attention due to its minimally invasive nature and promising initial outcomes. The overall data are still very limited. Thus, we report the long-term outcomes for Asian patients who underwent G-POEM, focusing on the predictive factors of clinical success.

## 2. Patients and Methods

### 2.1. Study Design and Inclusion Criteria

This study is a retrospective study that was designed to analyse data from 16 consecutive patients who underwent G-POEM performed by an expert endoscopist for refractory gastroparesis between August 2016 and October 2017 at Zhongshan Hospital. The inclusion criteria were as follows: (1) patients who were older than 18 years old; (2) patients who had gastroparesis based on the presence of symptoms, including postprandial fullness/early satiety, nausea/vomiting, and bloating, and who had a score ≥20 on the Gastroparesis Cardinal Symptom Index (GCSI) (Table 1) and evidence of impairment in gastric emptying scintigraphy (GES) (either a delay in the half emptying time or a retention percentage >60% at 2 hours); and (3) patients who were refractory to conservative treatment, including dietary modification and drug therapy with prokinetics and antiemetics. All patients received conservative treatment and showed minimal response to traditional medical therapy. Medication, such as proton pump inhibitors (PPIs), metoclopramide, mosapride, and domperidone, was given to all patients (100%), and a nasojejunal nutrition tube was placed in five patients (31.3%).

GES was performed using 50 g of bread marked with a nuclear tracer, and patients were scanned in the supine position. However, 2 patients completed GES in a semireclining position to avoid aspiration because they vomited violently after lying down.

The exclusion criteria included patients younger than 18 years old, patients whose endoscopy showed peptic ulcer or gastric outlet obstruction (tumour, fibrosis, etc.), and patients who had GCSI<20, normal GES, or any contraindication to G-POEM or anaesthesia.

The study was approved by the Ethics Committee of Fudan University. Written informed consent was obtained before the endoscopic procedure. Patient demographics and related medical history data (aetiology of gastroparesis, gastroparetic symptoms, GCSI score, results of GES, procedure details (length of tunnel and myotomy, closure method, and procedure time), adverse events, duration of hospital stay, and follow-up (GES and GCSI score)) were evaluated.

### 2.2. G-POEM Procedure

The G-POEM procedure was performed with single-channel gastroscopy or dual-channel endoscopy (GIF-H260 or GIF-2T240, Olympus). To provide a better view of the submucosal layer, a transparent cap (D-201-11802, Olympus) was attached to the tip. Other equipment included a hybrid knife (T-type, Erbe, Germany), hot biopsy forceps (FD-410LR, Olympus), clips (HX-610-90, HX-600-135, Olympus; ROCC-D-26-195, Micro-Tech, Nanjing, China), an OverStitch suturing device (Apollo Endosurgery, Austin, Texas), and a high-frequency generator (VIO-200, Erbe, Germany).

**Figure 1:**
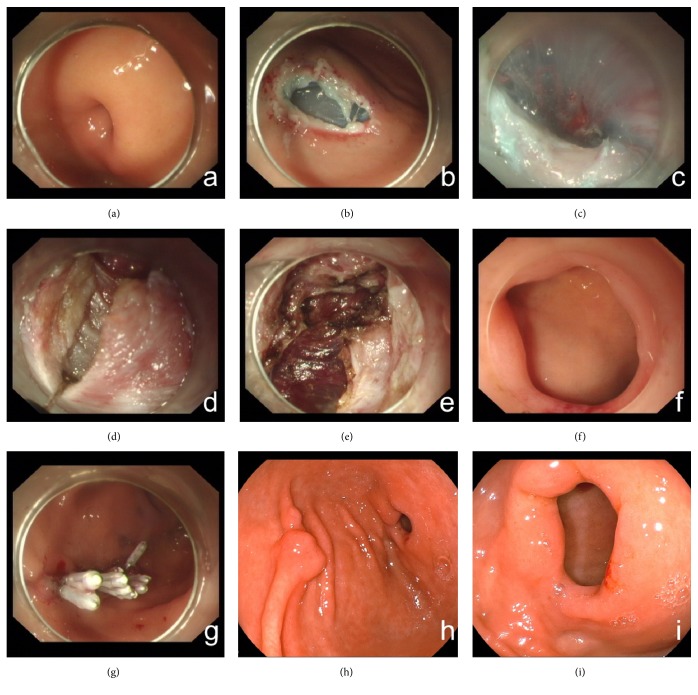
G-POEM procedure: (a) endoscopic view of the narrow pylorus; (b) injection and submucosal incision made 3 to 4 cm from the pylorus in the greater curvature of the antrum; (c) tunnel creation to approximately 2 cm passing over the pylorus; (d)-(e) myotomy of the pyloric and antral muscular layers; (f) endoscopic view of the pylorus after G-POEM; (g) mucosal entry closure by metal clips; (h) mucosal entry scar after 3 months of healing; and (i) endoscopic view of the pylorus after 3 months.

All patients were treated under general anaesthesia with tracheal intubation. The G-POEM procedure was performed as follows. (a) A submucosal cushion was created by submucosal injection (a mixed solution of 100 ml saline, 1 ml indigo carmine, and 1 ml epinephrine). (b) A mucosal incision was made 5 cm from the pylorus in the greater curvature of the stomach. (c) A submucosal tunnel was created from the mucosal entry to approximately 1 cm over the pylorus. (d) Full-thickness myotomy was performed from 2 to 3 cm proximal to the pylorus to 1 cm beyond the duodenal bulb. (e) After haemostasis, the mucosal entry was closed with metal clips or a suturing device (Figure 1). The mucosal incision was closed by metallic clips in thirteen patients (81.2%) and by an endoscopic suture device (OverStitch) in three patients (18.8%). During the procedure, mucosal injury occurred in three patients and was closed completely by metallic clips.

### 2.3. Perioperative Management

Before the G-POEM procedure, routine blood tests, including complete blood counts and coagulation profiles, and electrocardiography were obtained to evaluate the patient's general condition. PPIs and intravenous antibiotics were administered conventionally to all patients after G-POEM. The patients' abdominal symptoms and signs were carefully monitored during the postoperative period. Patients were discharged on PPIs for two months.

### 2.4. Follow-Up

Gastric emptying function was assessed by GES in our hospital three months after G-POEM, and the patients' symptoms were measured by GCSI after the procedure (at one month, three months, and every 6 months thereafter). The first endoscopic follow-up was arranged within 3 months after G-POEM, and the procedure was repeated annually thereafter to observe wound healing and monitor potential reflux.

### 2.5. Outcome Parameters

The primary outcome parameter was an assessment of the clinical efficacy of G-POEM, which was defined by symptomatic improvement of the patient and* a significant drop in the GCSI *at least > 50% of the baseline and without the need for further hospitalizations for nutritional support [11].

The secondary outcome parameter was the identification of possible predictive factors for clinical success and the detection of possible cut-off values for such predictive factors.

## 3. Results

This study included a total of sixteen patients; 11 (68.7%) patients were males, and 5 (31.3%) patients were females. Their ages ranged from 26 to 82 years, with a median age of 63.5 years. Thirteen patients were postsurgical (81.2%), and three patients had diabetes (18.8%). The patients' demographic data and clinical features are shown in Table 2.

**Figure 2:**
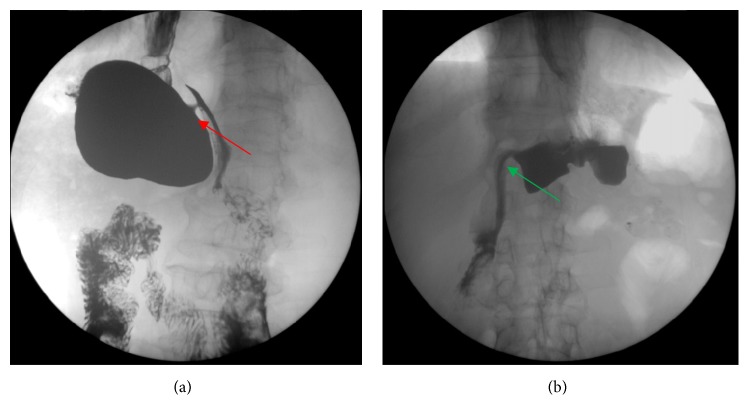
(a) Upper gastrointestinal radiography before G-POEM showing a narrow pyloric canal (shown with a red arrow) in the patient post-subtotal gastrectomy and (b) upper gastrointestinal radiography showing contrast passing freely through the pylorus after G-POEM (shown with a green arrow).

Before the G-POEM procedure, all patients suffered from severe nausea/vomiting, fullness/early satiety and bloating. The GCSI score ranged from 20 to 38, with a mean (±SD) of 24.25 (±5.47). Early satiety, vomiting, and nausea were the top three predominant symptoms, with mean scores of 4.06±1.18, 3.75±1.61, and 3.37±1.59, respectively, as shown in Table 2. Patients who underwent proximal subtotal gastrectomy were recommended for upper gastrointestinal radiography, and a narrow pyloric canal was found in all patients (Figure 2).

The GES half emptying time ranged from 102.2 to 328.9 min, with a mean (±SD) of 183.20±77, while the mean retention time at 2 hours ranged from 60.2% to 92%, with a mean (±SD) of 69.33±11.46%.

The G-POEM procedure was achieved successfully in all patients (100%), with a mean procedure time of 45.25±12.96 min. No severe intraoperative adverse events, including significant bleeding or pneumoperitoneum, occurred. The median hospital stay was 6 days (range: 4-10 days). No procedure-related adverse events, such as mucosal tears, delayed bleeding, or perforation, were observed. Only one patient developed pyloric stenosis secondary to scar formation on the 45^th^ day, and he underwent endoscopic radial incision (ERI).

### 3.1. The Primary Outcome Parameter

Clinical response was assessed in all patients during a median follow-up of 14.5 months (range: 5-19 months). There were significant decreases in GCSI scores, mean half emptying time and 2-hour retention after G-POEM compared to the baseline values, with* P* values of <0.0001, 0.012, and 0.012, respectively (Table 3).

Thirteen (81.25%) patients showed a substantial long-term clinical improvement in symptoms related to delayed gastric emptying and GCSI scores.

**Figure 3:**
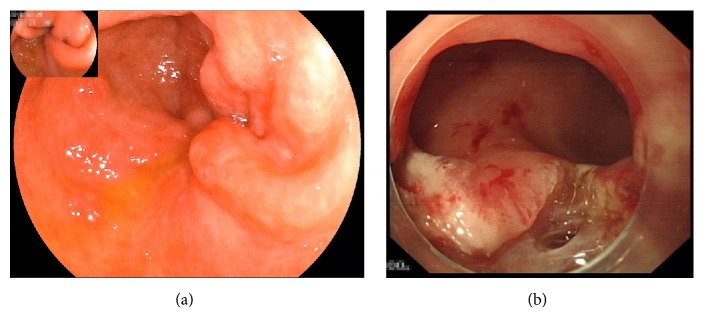
(a) Endoscopic view of a recurrent patient showing the mucosa near the pylorus shrinking and the scar beneath contracting and (b) pylorus opened widely after endoscopic radial incision.

Among the three patients who failed to experience a long-lasting improvement, one patient developed persistent vomiting on the 45^th^ day after the procedure, as mentioned above. Gastroscopy was performed and revealed pyloric stenosis secondary to scar formation. The patient received a salvage ERI and was discharged successfully after semifluid diet was allowed (Figure 3). The other two patients had suboptimal responses to G-POEM; both patients had a long-standing history of uncontrolled diabetes and were previously diagnosed with diabetic peripheral neuropathy. One patient showed little early response to G-POEM at the first follow-up (three months after the procedure); the GCSI score was 34 before the procedure and 28 after the procedure. The other patient showed marked improvement, with a GCSI score of 22 before the procedure and 10 after the first follow-up, at which he had gained approximately 10 kg in weight. However, he developed worsening of symptoms 7 months after G-POEM, and his GCSI started to increase again, reaching 19. Both patients were readmitted to the hospital for nutritional support.

### 3.2. The Secondary Outcome Parameter

Univariate regression analysis showed significant positive correlations of GCSI score and GES values with unfavourable future clinical outcomes of the patients (Table 4). The predictive factors identified in the univariate analysis were not confirmed in the multivariate analysis.

**Figure 4:**
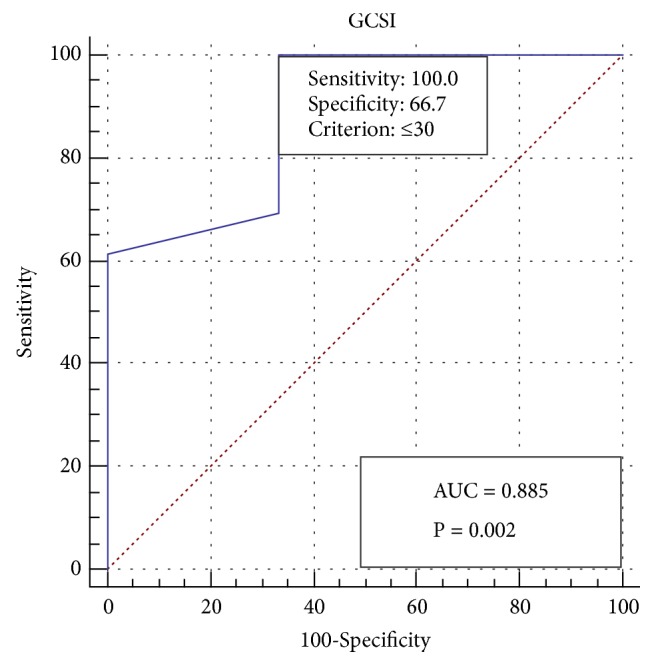
Receiver operating characteristic (ROC) curve showing 100% sensitivity and 66.7% specificity of the Gastroparesis Cardinal Symptom Index (GCSI) at a cut-off value ≤30 for predicting the clinical response of the patients.

**Figure 5:**
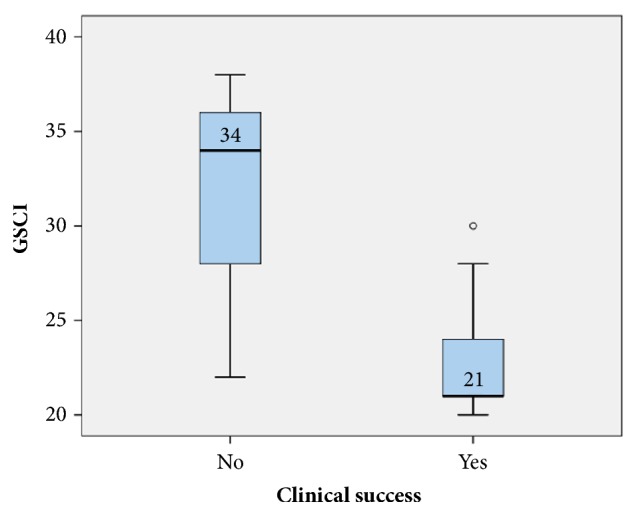
Box plot of the Gastroparesis Cardinal Symptom Index (GSCI) among patients who achieved clinical success and patients who did not.

GCSI scores had a high sensitivity (100%) and specificity (66.7%) at a cut-off value of ≤30 for predicting favourable clinical outcomes in patients (Figure 4). GCSI scores have a high negative predictive value (NPV) of 100% and a positive predictive value (PPV) of 92.86% (Table 5). The box plot showed that GCSI scores in those who achieved clinical success ranged from 20 to 30, with a median of 21 and an interquartile range (IQR) of 4, while the GCSI scores of those who failed to achieve clinical success ranged from 22 to 38, with a median of 34 and an IQR of 8 (Figure 5).

**Figure 6:**
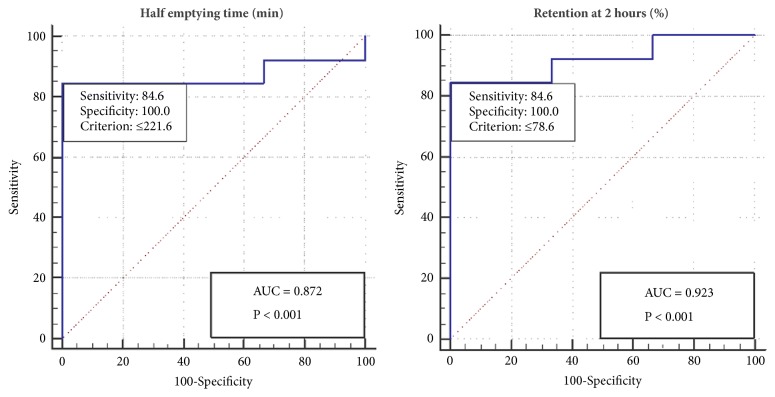
Receiver operating characteristic (ROC) curve showing 84.6% sensitivity and 100% specificity of gastric emptying scintigraphy (GES) for predicting the clinical response of the patients.

**Figure 7:**
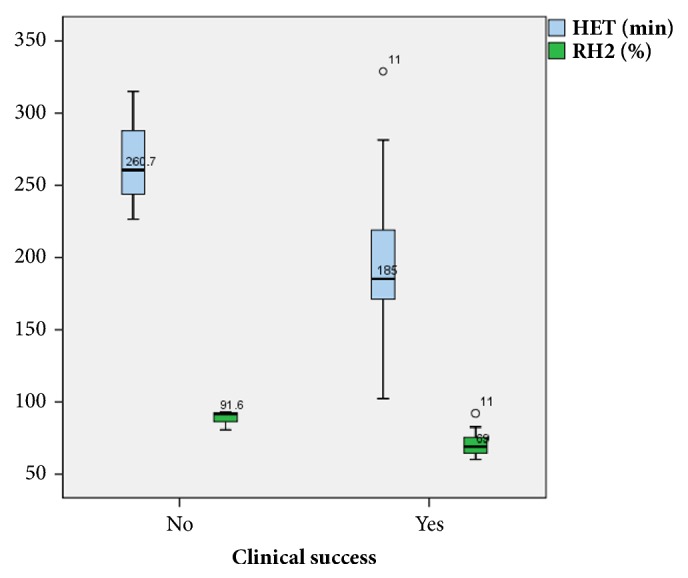
Box plot showing the distribution of gastric emptying scintigraphy (GES), including both half emptying time (HET) in minutes and retention percentage at 2 hours (RH2), among patients who achieved clinical success and patients who did not.

**Figure 8:**
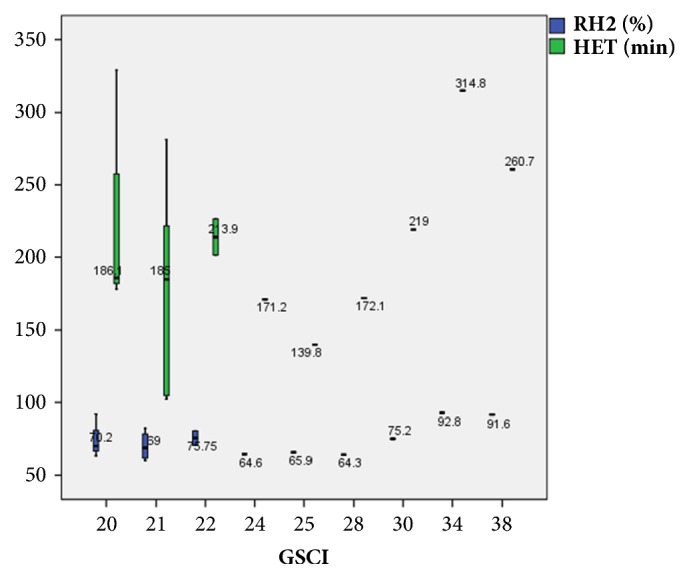
Box plot of the gastric emptying scintigraphy (GES) including the half emptying time (HET) and the retention percentage at 2 hours (RH2) against Gastroparesis Cardinal Symptom Index (GSCI).

In GES, the half emptying time and the 2-hour retention had high specificity (100%) and sensitivity (84.6%) at cut-off values of <221.6 minutes and 78%, respectively (Figure 6). GES had a high PPV (100%) and an NPV of 59.97% (Table 5). Box plots of the half emptying time and the retention percentage at 2 hours showed ranges from 102.2 to 328.9 min and 60.2% to 92%, respectively, with median values (IQRs) of 185 (64) and 69 (13), respectively, among patients who achieved clinical success. Additionally, among patients who failed to achieve clinical success, these values ranged from 226.4 to 314.8 min and 80 to 92.8%, respectively, with median values (IQRs) of 260.7 (44) and 91.6 (6), respectively (Figure 7). Box plots of the GES including the half emptying time and the retention percentage at 2 hours against GSCI are shown in Figure 8.

## 4. Discussion

Gastroparesis is a clinical syndrome that is characterized by a delay in gastric emptying in the absence of true mechanical obstruction [12]. Gastric emptying entails interactions among smooth muscle, enteric and extrinsic autonomic nerves, and the interstitial cells of Cajal (ICCs) [13]. The exact pathogenesis of gastroparesis is not fully understood; however, multiple hypotheses have attempted to explain its pathogenesis. Autonomic neuropathy, gastric hypersensitivity, vagal nerve dysfunction impairing pyloric relaxation, loss of expression of neuronal nitric oxide synthase, and loss of ICCs are the most recent and most common hypotheses [14, 15]. Accordingly, the treatment of gastroparesis is usually directed towards targeted pathogenesis. Pharmacotherapy in the form of prokinetics and selective motilin and ghrelin agonists is the mainstay of treatment. However, pharmacotherapy has limited efficacy and undesirable side effects, including some side effects that are irreversible [16]. Gastric electrical stimulation has been reported in several studies [17, 18] and has shown some benefits, especially in relieving nausea/vomiting symptoms; however, the use of this approach is limited by uncontrolled trials and the high cost of surgical devices.

Recent therapeutic options have focused on relieving pylorospasm, which presents with increased pyloric tone and phasic contractions. These therapies include botulinum toxin injection, endoscopic transpyloric stent placement and fixation, and laparoscopic pyloroplasty. Several open-labelled studies on botulinum toxin injection [19–22] observed improvements in gastric emptying and symptoms for several months. However, two randomized double-blind placebo-controlled studies [20, 21] found no specific improvement in symptoms compared with placebo (saline solution injection). Nevertheless, botulinum toxin injection is still regarded as a screening tool for patients who respond to therapies targeting pyloric and antral hypertonicity. Endoscopic transpyloric stent [23] and laparoscopic pyloroplasty [24] are two promising techniques associated with statistically significant reductions in symptoms. A long-term study [25] included 177 patients who underwent laparoscopic pyloroplasty over a period of five years and showed an 86% improvement in GES, with normalization in 77% of patients. Unfortunately, this technique still requires surgical abdominal entry with potential adverse events, including leakage, bleeding, and wound infections.

Building on the concept of relieving pylorospasm, endoscopic pyloromyotomy (G-POEM) evolved and is becoming a potential viable alternative for the treatment of gastroparesis. Herein, we present a series of Asian patients who were treated by this novel technique with encouraging outcomes. The mean procedure time was 45.25±12.96 min, which is consistent with most previous studies [7, 9, 11]. However, Xue et al. [26] stated that the use of fluoroscopic guidance with the placement of an endoclip on the pylorus at the 9 to 11 o'clock position to facilitate identification (a challenging step in G-POEM) significantly shortened the procedure time by approximately 36±13 min.

Technical success was achieved in all patients, and this finding is consistent with the results of many studies that reported the safety of the procedure. Questions remain concerning the efficacy of the procedure. We report an 81.25% clinical success rate with a mean follow-up period of 14.5 months. Gonzalez et al. showed success rates of 79% and 70% for periods of 3 and 6 months, respectively [27]. Additionally, a recent study showed a success rate of 81% with a follow-up of 6 months, which could represent a promising outcome for those patients [28].

In our study, we found that GCSI and GES showed significant improvements before and after procedure. Nevertheless, these parameters could also be used as predictive measures of clinical success after the procedure, with high sensitivity and NPV for the GCSI, which indicates that it is a good negative test at a cut-off value of ≤30. The GES appears to be a good positive test, with a high specificity and PPV for predicting clinical success based on both the half emptying time and the retention at 2 hours. Currently, there are no reliable ways to predict which patients will respond to G-POEM. A recent study that focused on the predictive factors for clinical response found that female gender and diabetes were predictors of failure after G-POEM [27].

The reasons for unresponsiveness remain unclear. The cause of gastroparesis has been investigated, especially in patients with diabetes [29, 30]; however, only 3 diabetic patients were included in our study, and this number would not lead to clinical significance.

In conclusion, this study presents a promising long-term clinical outcome for G-POEM, with a high technical success rate and few adverse events. GCSI score and GES values could be used as predictors of favourable outcomes. Prospective multicentre randomized controlled trials are still needed to confirm these findings and determine the exact factors that predict patient responsiveness to G-POEM.

## Figures and Tables

**Table 1:** Gastroparesis Cardinal Symptom Index (GCSI).

	**None **	**Very mild**	**Mild**	**Moderate**	**Severe**	**Very severe**
**Nausea (feeling sick to your stomach as if you were going to vomit or throw up)**	0	1	2	3	4	5

**Retching (heaving as if to vomit, but nothing comes up)**	0	1	2	3	4	5

**Vomiting**	0	1	2	3	4	5

**Stomach fullness**	0	1	2	3	4	5

**Not able to finish a normal-sized meal**	0	1	2	3	4	5

**Feeling excessively full after a meal**	0	1	2	3	4	5

**Loss of appetite**	0	1	2	3	4	5

**Bloating (feeling like you need to loosen your clothes)**	0	1	2	3	4	5

**Stomach or belly visibly large**	0	1	2	3	4	5

**Table 2:** Demographic features, clinical features, and follow-up.

**Age (years, median (range))**	63.5 (26-82)
**Gender (ratio, male/female)**	11/5
**Aetiology of gastroparesis**	
Postsurgical	13 (81.2%)
Diabetic	3 (18.8%)
**Duration of disease before G-POEM (months, median (range))**	22 (2-84)
**Previous therapy**	
PPIs	16 (100%)
Metoclopramide	14 (87.5%)
Nasojejunal nutrition tube	5 (31.3%)
**GCSI score (mean (±SD)) before G-POEM**	
Nausea	3.37±1.59
Retching	2.56±1.82
Vomiting	3.75±1.61
Stomach fullness	3.13±1.67
Not able to finish a normal-sized meal	4.06±1.18
Feeling excessively full after a meal	3.31±1.54
Loss of appetite	1.94±1.98
Bloating	1.94±1.88
Stomach or belly visibly large	0.19±0.75
Total	24.25±5.47
**Gastric emptying scintigraphy before G-POEM**	
Half emptying time (min, mean (±SD))	183.20±77.39
Retention at 2 hours (mean (±SD))	69.33±11.46%
**Technical success rate**	100%
**Procedure time (min, mean (±SD))**	45.25±12.96
**Closure of mucosal incision**	
Metal clip	13 (81.2%)
Overstitch	3 (18.8%)
**Hospital stay (days, median (range))**	6 (4-10)
**Intraoperative adverse events**	0
**Follow-up time (months, median (range))**	14.5 (5-19)
**Total GCSI score (mean (±SD)) after G-POEM**	6.37±6.34
**Gastric emptying scintigraphy (n=8) after G-POEM**	
Half emptying time (min, mean (±SD))	83.98±34.65
Retention at 2 hours	33.38±18.17%
**Long-term clinical remission**	13 (81.3%)

G-POEM: gastric per oral endoscopy myotomy, GSCI: Gastroparesis Cardinal Symptom Index.

**Table 3:** Patient assessments before and after G-POEM.

	**Before G-POEM **mean (±SD)	**After G-POEM **mean (±SD)	***P* value**
**GCSI**	24.25±5.47	6.37±6.34	<0.001
**GES**			
Half emptying time (min)	183.20±77.39	83.98±34.65	0.012
Retention at 2 hours (%)	69.33±11.46%	33.38±18.17%	0.012

G-POEM: gastric per oral endoscopy myotomy, GSCI: Gastroparesis Cardinal Symptom Index, GES: gastric emptying scintigraphy.

**Table 4:** Univariate regression analysis of the clinical outcomes of the patients.

	**Coefficient**	**Odds Ratio**	**95**%** CI**	***P* value**
**Age**	0.11	1.1168	0.99-1.25	0.017
**Disease duration**	0.076	1.07	0.93-1.24	0.1255
**GCSI**	-0.3	0.74	0.54-1.0037	0.0157
**Half emptying time**	-0.021	0.971	0.95-1.0042	0.059
**Retention at 2 hours**	-0.204	0.815	0.65-1.008	0.0078

GSCI: Gastroparesis Cardinal Symptom Index.

**Table 5:** Predictive values for successful clinical outcomes in the patients.

	**Sensitivity**	**Specificity**	**AUC**	***P* value**	**PPV**	**NPV**
**GSCI**	100%	66.7%	0.885	0.002	95.3%	100%
**Half emptying time (min)**	84.6%	100%	0.872	<0.001	100%	59.97%
**Retention at 2 hours (**%**)**	84.6%	100%	0.923	<0.001	100%	59.97%

GSCI: Gastroparesis Cardinal Symptom Index.

## Data Availability

The data belongs to the Endoscopy Research Centre in Zhongshan Hospital and a permission is requested to make it freely available.
